# Adolescent Health on Social Media and the Mentorship of Youth Investigators: Five Content Analysis Studies Conducted by Youth Investigators

**DOI:** 10.2196/29318

**Published:** 2021-09-15

**Authors:** Maggie Bushman, Shreya Godishala, Reese Hyzer, Joshua Jerisha, Anna Jolliff, Ethan Kaji, Bradley Kerr, Anjali Mathur, Owen Tsao

**Affiliations:** 1 Department of Pediatrics School of Medicine and Public Health University of Wisconsin - Madison Madison, WI United States; 2 Edgewood High School Madison, WI United States; 3 Madison West High School Madison, WI United States; 4 James Madison Memorial High School Madison, WI United States

**Keywords:** social media, anxiety, depression, self-esteem, Instagram, Reddit, Twitter, YouTube, content analysis, adolescent

## Abstract

Although the literature on adolescent health includes studies that incorporate youth perspectives via a participatory design, research that is *designed, conducted,* and *presented* by youth remains absent. This paper presents the work of 5 youth investigators on the intersecting topics of adolescent health and social media. Each of these youths was equipped with tools, knowledge, and mentorship for scientifically evaluating a research question. The youths developed a research question that aligned with their interests and filled a gap that they identified in the literature. The youths, whose projects are featured in this paper, designed and conducted their own research project, drafted their own manuscript, and revised and resubmitted a draft based on reviewer input. Each youth worked with a research mentor; however, the research questions, study designs, and suggestions for future research were their own.

## Introduction

### No Research About Us Without Us

Most scientists who work with human subjects are familiar with this tenet, which implies that study participants, including the youth, should not be treated merely as *passive subjects and beneficiaries* of research but rather as active contributors to the research process [1]. Indeed, a growing body of research suggests that youths and their communities alike benefit from youth participation in research [2-4]. However, one systematic review of youth participatory action research found that youths are seldom engaged in the earliest phases of the research process, including the assessment of needs and formation of research questions [3]. Another systematic review on youth participatory action research found that none of the 45 studies reviewed included youth as authors [4]. Thus, despite increased recognition of the need for youth inclusion in research, instances of youth defining research questions and authoring empirical manuscripts are rare.

In June 2020, partially in response to the COVID-19 pandemic, the *Journal of Adolescent Health* published a call to “transform the way that young people engage with designing and implementing adolescent health programs and policies” [5]. This empirical compilation extends this prerogative to suggest that youth should also be invited to author research. This suggestion may raise additional considerations and concerns. For example, teaching and mentoring youth through the basics of the research process is time-intensive, and many research teams do not prioritize the allocation of staff toward training youth. In addition, youths are often unfamiliar with processes that the scientific community has deemed crucial to legitimate scientific contributions, and thus, research produced by youth may differ from that produced by scholars with more training and resources. In this study, given the youths’ early stage in their research careers, certain expectations of typical empirical research articles were adjusted. For example, the youths received permission from mentors to collect smaller sample sizes, use simpler analytic approaches, and retain a few instances of less-scientific language to allow their unique voices to be present. These approaches are similar to professional researchers’ early investigations or pilot work, which often focus on detecting early findings to fuel larger hypothesis-driven studies [6,7]. We suggest that the need for adjustments such as these is not a sufficient reason to exclude youth from publishing research; rather, youths’ developmental and educational stages should be considered as important context for evaluating their work.

This compilation of youth research presents the work of five young investigators; 4 of the youth authors were in high school at the time of writing (JJ, EK, AM, and OT), and one was in her first semester of college (SG). All youth participated in the Summer Research Scholars (SRS) program, a program that uses a tested and empirically supported curriculum to guide adolescents through the steps of the research process to complete and present their own independent research project [8]. The youths whose projects were featured here experienced 3 months of training in the SRS program. They were provided with tools, knowledge, mentorship, and supervision to scientifically evaluate a research question. They selected their questions through a review of the literature, incorporation of their own areas of interest, and discussions with their peers and mentors. The youths then had approximately 6 months to design and conduct their own research project, draft their own manuscript, and revise and resubmit that draft based on reviewer input. Although each youth worked with a research mentor, the research questions, study designs, and suggestions for future research are their own.

### Common Methods Across Projects

#### Natural Language Processing

In this compilation, two of the five studies used natural language processing to evaluate the text. For these analyses, the Linguistic Inquiry and Word Count (LIWC) program was used [9]. This software program analyzes bodies of text for the frequency of keywords associated with psychologically meaningful categories, including thinking styles, attentional focus, and emotionality in a variety of experimental settings. LIWC builds on several decades of research to understand narrative voice in health [10-15] and uses validated internal dictionaries developed by a rigorous process—in which groups of judges reviewed 2000 words or word stems and determined how the reviewed words related to specific categories (eg, word count, total first-person usage, and negative emotion). During the LIWC analysis of a document, every word is compared with *dictionaries* of up to 74 dimensions across these categories. LIWC calculates the proportion of words falling into different categories, ranging from emotional words to words about social context [16]. LIWC has been validated for content and construct validity [11,17]. Interrater reliability discrimination of categories has been found to range from 86% to 100% depending on the dimension being assessed, supporting content validity.

#### Content Analysis

All five studies included content analyses as all or part of the approach. Content analysis is a systematic technique for developing categories into which data are sorted based on explicit rules [18]. Content analysis allows for the examination and quantification of social media content, such as original posts (text, images, and videos) and engagement with such posts (likes, comments, and shares). Content analysis has previously been used to evaluate discussions of health-related topics on social media [19,20]. Content analysis may produce more objective data on social media behavior than, for example, self-reports or interviews, which may be vulnerable to social desirability or recall bias. Content analysis approaches allow for the testing of hypotheses through the development of a deductive codebook based on theory or clinical guidelines. Content analysis also allows for the exploratory evaluation of novel phenomena through the inclusion of inductive codes. Furthermore, the content analysis of publicly available social media data is often granted exempt status by institutional review boards, as was the case with each of these projects. This research method allowed adolescents to conduct research on an accelerated timeline.

The studies below are grouped by method, with the two studies using natural language processing appearing first, followed by the three studies that used content analysis alone. For each study, the youth authors are listed first, and the second author is the adult research mentor.

## Study 1

*This study was prepared by Joshua Jerisha and mentor Reese Hyzer*

### COVID-19, Social Distancing, and Adolescent Mental Health on Twitter: Web-Based Content Analysis

#### Introduction

##### Background

As of February 26, 2021, the COVID-19 pandemic was associated with more than 2.5 million deaths globally, with 508,127 confirmed deaths in the United States alone [21]. Nonpharmaceutical interventions have been used to curb the spread of the virus. Social distancing measures (physical distancing, quarantines, and remote work or school) have been found to be one of the most effective methods for reducing COVID-19 transmission [22], and in compliance with these measures, many communications have shifted to a virtual format. In turn, social media may play a significant role in communication regarding the risks associated with COVID-19 and social distancing measures.

In the early days of the first outbreak (January 31, 2020, to February 2, 2020), social media use among Chinese citizens aged 18 years or older was correlated with a 22.6% increase in anxiety and a 48.3% increase in depression [23]. Before the pandemic, anxiety and depression were not uncommon among adolescents, with 16.5% of US youth aged 6-17 years experiencing a mental health condition in 2016 [24]. In 2020, 98.1% of US adolescents reported compliance with social distancing [25]. Adolescents may be at a unique risk for mental health challenges because of a combination of social media use, prepandemic rates of anxiety and depression, and reduced social contact.

The World Health Organization (WHO) declared COVID-19 a global pandemic on March 11, 2020. By April 2, 2020, the WHO launched their #HealthyAtHome campaign, followed by advice on the use of face masks by April 6, 2020. All these announcements were communicated in a tweet from the WHO [26].

##### Objectives

Currently, associations among social media use, mental health, and the language surrounding COVID-19–related posts remain unclear. The shifting state of the institutional response to COVID-19 may be reflected in the broader discussion of the pandemic on Twitter. Thus, changes in information found on social media may be associated with related shifts in mental health. In light of this, our study aims to explore the independent and co-occurring mentions of social distancing and mental health on Twitter between March and April 2020, as well as the content and language featured in these posts.

#### Methods

##### Study Design

We conducted an exploratory content analysis and linguistic analysis of Twitter. Our social media unit of analysis was the *tweet*, which we defined as a single post created by a user account containing between 1 and 280 characters of text. This study was exempt from human subjects review by the University of Wisconsin-Madison Institutional Review Board.

##### Search and Sampling Strategy

We sought to obtain a representative sample of COVID-19–related tweets from March 2020 to April 2020. We manually sampled posts from the *Top* category of a custom Twitter search for *#COVID19* from both months. We selected the first 50 tweets from the list for each month. Twitter’s proprietary sorting algorithm governs the retrieval of tweets via this mechanism. This design sought to replicate how adolescents might encounter COVID-19–related tweets available to the general public. Tweets favored by the Twitter algorithm are given more exposure on the platform and thus are most relevant to the average adolescent Twitter user.

##### Social Media Inclusion Criteria

Sampled tweets were included for analysis if they contained English language text and the hashtag #COVID19. If either of these criteria were not met, the corresponding tweet was excluded from the analysis. Similarly, duplicate tweets were eliminated from the sample set.

##### Measures

Our study examined the following three categories of data: descriptive profile data, post content, and LIWC scores.

###### Descriptive Profile Data

Descriptive profile data for each tweet included its post date, number of likes, number of account followers, and account verification status. These data points offer context regarding the size of the audience and the types of accounts posting the tweets. Verified accounts usually belong to users of public interest, ranging from celebrities to institutions such as the WHO and accredited professionals, including epidemiologists.

###### Post Content

Post content data consisted of the multimedia attached to the tweet in the form of a hyperlink, image, or video, as well as references to social distancing and mental health.

Multimedia data were recorded to provide context for social media engagement (interaction with posts through likes, comments, and other means), and by extension, its weighting by the algorithm. In previous research, social media users reported that they would preferably engage with posts containing an image (68%), a video (50%), and a hyperlink (16%) [27]. Thus, greater multimedia presence indicates more relevance to typical adolescent users because of the relationship between high social media engagement and Twitter’s algorithm.

References to social distancing and mental health were based on keywords established in the codebook, which are defined in Table 1.

**Table 1:** Study 1—social distancing and mental health reference coding criteria and results for March and April tweets (N=100).

Reference	Codebook keywords	Example tweet verbatim	March tweets (n=50), n (%)	April tweets (n=50), n (%)
Social distancing	*Social distancing*, *physical distancing*, *stay home*, *shelter in place*, *lockdown*, *shutdown*, *quarantine*, *isolation*, *self-isolate*, *remote learning*, *remote work*, and *flatten the curve*	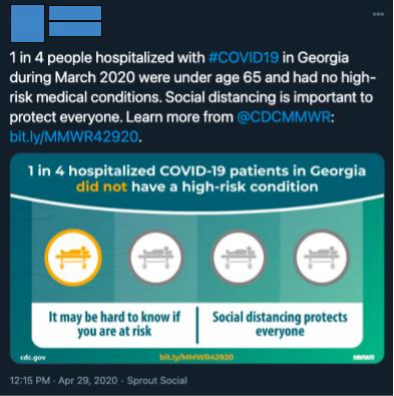	11 (22)	21 (42)
Mental health	*Mental health*, *anxiety*, *depression*, *anxious*, *depressed*, *stressed*, *frustrated*, *angry*, *sad*, *scared*, and *afraid*, *resilient*	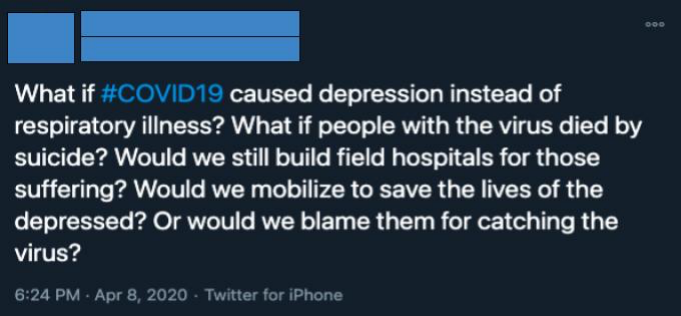	13 (26)	5 (10)
Both	One or more keywords from social distancing and mental health	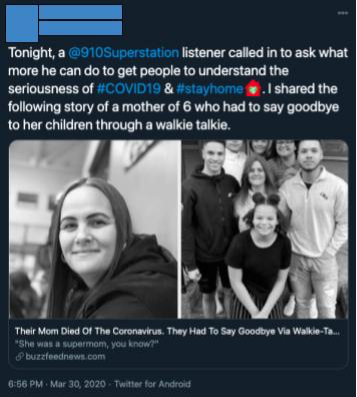	11 (22)	6 (12)

###### The LIWC Program

We used the most recent version of the LIWC program, which is a text analysis program described in the Introduction section of this compilation [28]. LIWC has previously been used in studies on news media coverage of cyberbullying [29], gender differences in pediatric residency personal statements [30], and linguistic convergence among friend groups [31]. Output variables, referred to as *LIWC scores* in this paper, represent the frequency of keyword occurrence. Each of these numerical scores can be compared between datasets to illustrate the relative trends in the written materials. The only LIWC score that differs from this design is *emotional tone*, which is evaluated as a percentile between 0% and 100% [28]. Sample keywords from LIWC dictionaries are listed in Table 2. Our LIWC dictionaries of interest were *anger* and *sadness* (emotions), *anxiety* and *risk* (perceptions), and *first-person singular* and *first-person plural* (pronouns). The *emotions* and *perceptions* dictionaries correspond to several relevant components of adolescent mental health (eg, symptoms of stress, anxiety, and depression), whereas the singular and plural *pronoun* dictionaries provide insight into the framing of tweets. All these factors help inform our understanding of the possible mental health implications for adolescent users.

**Table 2:** Study 1—Linguistic Inquiry and Word Count (LIWC) dictionary, example terms, and LIWC scores for March and April tweets with two-tailed t test results.

LIWC dictionary	LIWC example terms	LIWC scores		*P* value (adjusted)
		March tweets, mean (SD)	April tweets, mean (SD)	
Emotional tone	N/A^a^	50.25 (37.85)	38.98 (33.04)	.12
I	*I*, *me*, and *mine*	2.23 (4.32)	0.77 (1.66)	.03
We	*We*, *us*, and *our*	1.49 (2.56)	1.61 (2.39)	.80
Anxiety	*Worried* and *fearful*	0.67 (1.33)	0.75 (1.41)	.77
Anger	*Hate*, *kill*, and *annoyed*	0.68 (1.52)	0.49 (1.27)	.51
Sadness	*Crying*, *grief*, and *sad*	0.77 (1.76)	0.77 (1.85)	.99
Risk	*Danger* and *doubt*	0.99 (1.74)	1.60 (2.51)	.16

^a^N/A: not applicable.

##### Data Collection Procedures

Data collection began with Twitter’s *Advanced search* under the *Search filters* section of the website. This type of search allows a user account to filter tweets using hashtags and dates. *#COVID-19* was input into the hashtag field, with *March 1, 2020*, as the start date and *March 31, 2020,* as the end date for March. For April, we switched the data parameters to *April 1, 2020*, for the start date and *April 30, 2020*, for the end date. Age-related filters were not available, so our selected tweets were produced by accounts associated with organizations and individuals of various ages. Tweet text verbatim was copied into a spreadsheet before the number of the tweet’s likes, the number of account followers, and account verification status were recorded. Tweet text was analyzed using the LIWC program to determine LIWC scores. We did not collect any personally identifiable data, such as full names or Twitter handles. Data were collected between July 22, 2020, and July 26, 2020.

##### Analyses

Data were separated by month to represent March 2020 and April 2020 to interpret our findings through comparisons. Descriptive statistics were conducted and included means and SDs for tweet likes and followers, percentages for account verification, and percentages for multimedia. A chi-square test was used to assess the relationship between the month and the proportion of mentions of social distancing, mental health, and both or none. Independent *t* tests were conducted to compare LIWC scores between tweets posted in March and April. Statistical analyses were conducted using the software STATA 15.1 (StataCorp LLC), and statistical significance was set at *P*<.05.

#### Results

##### Descriptive Profile Data

We identified a national sample of 100 tweets associated with the COVID-19 pandemic, with 50 sourced from March 2020 and another 50 sourced from April 2020. The March tweets averaged 586.3 likes (SD 1950.7) and 100,103.5 account followers (SD 167,936.8), whereas the April tweets averaged 358.5 likes (SD 550.3) and 482,775.2 account followers (SD 1,208,351). Of the 50 tweets sampled, 31 (62%) from March and 30 (60%) from April were posted by verified accounts.

##### Post Content

Among the 50 March tweets, 9 (18%) contained a hyperlink, 20 (40%) contained an image, 8 (16%) contained a video, and 13 (26%) contained no media. Among the 50 April tweets, 12 (24%) contained a hyperlink, 23 (46%) contained an image, and 6 (12%) contained a video, whereas 9 (18%) did not contain any media. We found that the frequency of social distancing mentions was greater in April tweets (21/50, 42%) than in March tweets (11/50, 22%; *P*=.04). There were more mental health mentions in March tweets (13/50, 26%) than in April tweets (5/50, 10%; *P*=.04). Tweets that included mentions of both social distancing and mental health were present more in March tweets (11/50, 22%) than in April tweets (6/50, 12%; *P*=.04). Finally, tweets that mentioned none of these categories were less prevalent in March tweets (15/50, 30%) than in April tweets (18/50, 36%; *P*=.04; see Table 1 for further details).

##### LIWC Results

An independent sample *t* test showed that March 2020 tweets had significantly more singular (I) pronouns (mean 2.23, SD 4.32) than April 2020 tweets (mean 0.77, SD 1.66; *t_98_*=2.24; *P*=.03). All other results were statistically insignificant (*P*>.05). The complete list of LIWC scores and *t* test results are listed in Table 2.

#### Discussion

##### Principal Findings

We found that references to social distancing increased, whereas references to mental health and both categories decreased between March and April. In addition, *first-person singular* pronoun usage decreased significantly during the same timeframe. On the basis of these findings, we drew cautious inferences regarding adolescent mental health amid the COVID-19 pandemic.

##### Social Media Discourse

Our results suggest that social distancing may have become a more salient topic of social media discourse as the pandemic progressed. In the early months of the crisis, fears surrounding the coronavirus remained relatively consistent, supported by the lack of significant differences between the March and April data sets of *anxiety* and *risk*. In addition, social distancing remained prominent between both months, whereas references to mental health decreased from March to April. These findings may provide some insight into adolescent mental health amid the COVID-19 pandemic. Previous studies have shown that adolescents are especially sensitive to changes in social stimuli, particularly in the reduction of interaction with peer groups [32]. References to social distancing may emphasize this unfulfilled need. Adolescents who increasingly come into contact with social media content involving social distancing may be at an elevated risk of mental health consequences.

##### Individualism Versus Collectivism

The decrease in the prevalence of *I* pronouns suggests less frequent usage of an individualistic language as the pandemic progressed. However, our findings do not support an increase in the prevalence of *we* pronouns. One possible explanation is a shifting focus to more factual and objective content phrased in the third person (eg, public health statements). In the future, larger studies may be able to detect trends in language to examine changes in individualistic or collectivist sentiment over time or across groups. Collectivism emphasizes the priorities of the group over the individual [33], which may reduce the frequency with which individual mental health is referenced. For this reason, fewer references to mental health may indicate a societal paradigm shift away from the perspective of individuals. The collectivist perspective is associated with positive mental wellness under certain circumstances. In a study, cultural collectivism was correlated with a reduction in suicidal ideation among grieving women [32]. In contrast with the isolation associated with social distancing measures, a collectivist outlook may have a positive effect on adolescent mental health.

##### Limitations

A few considerations must be taken into account when interpreting the results of our study. The breadth of our content analysis provides insight into macrolevel social trends, although our sample size was small. For a few of our LIWC measures, statistical significance may have been achieved in a larger sample. Similarly, selected posts were prioritized by the Twitter algorithm rather than being produced or shared by the adolescent population. With these caveats in mind, our data can be used to draw cautious inferences regarding specific population segments, although they are not a perfect metric of individual attitudes and perceptions. In the future, surveys or focus groups can be used to corroborate the content analysis findings. In addition, the web-based or offline divide may have played a role in our study. Opinions expressed on the web can differ from personal beliefs offline. Interviews with adolescents would provide an understanding of offline perceptions of social distancing and mental health.

##### Conclusions

In light of our study, clinicians should consider the type of content being consumed on social media when addressing adolescents’ mental health during the COVID-19 pandemic. Mass media sources (television and newspaper stories) have the capacity to shape public responses to crises. Previous research has demonstrated that as few as 4.6% of these sources *express empathy* in response to crises [34]. Mass media messaging is related to social media, as Twitter serves as a platform that forwards these sources to a wider audience. When discussing mental health challenges, clinicians should ask their adolescent patients about the tone of the media they consume and how it affects their mood and thought processes. These considerations will be especially relevant in treating the long-term mental health consequences of the pandemic. Future studies should investigate the offline mental health implications of COVID-19 on social media using more targeted methods, such as interviews and focus groups. These studies can be regularly administered for a timeframe longer than 2 months to better track the evolution of the adolescent mental health response to COVID-19.

## Study 2

*This study was prepared by Shreya Godishala and mentor Anna Jolliff*

### Pro- and Antiopioid Posts on Reddit: Content Analysis

#### Introduction

##### Opioid Use Among Adolescents

In 2018, almost 70,000 people died from drug overdose [35]. Two out of three overdose deaths involved opioids such as prescription opioids, heroin, or synthetic opioids (eg, fentanyl). A large number of opioid users are adolescents [36]. Research suggests that 5.5% of those aged 17 years endorse opioid misuse [37]. Investigating opioid-related content on social media platforms commonly used by adolescents has the potential to reveal patterns of opioid abuse at a national scale, understand the opinions of adolescents and young adults, and provide insight to support prevention and treatment [38].

##### Social Media and Opiate Use

Despite the large number of adolescents who misuse opioids [36], little is known about how social media influences adolescents’ use or misuse of opiates. One study found an association between a participant tweeting about opioids and offline opioid overdoses [39]. Furthermore, previous research has shown that engagement with alcohol-related and e-cigarette–related social media is associated with more offline use of these substances [40,41]. Therefore, it is important to understand the messages that adolescents view on social media regarding the risky behavior of using opiates, as these messages may predict their behavior.

Reddit is a forum-based social media platform in which subcommunities, or *subreddits*, are built based on people’s interests [42]. A study on regular news consumers found that about half of Reddit users were young adults aged between 18 and 29 years [43]. To date, few empirical studies have discussed opioid use in Reddit communities. A previous study evaluated posts from anonymous and nonanonymous users in an opioid-related Reddit thread [44]. This study found that nonanonymous users were more likely to use words related to the past than anonymous users, who may have felt more comfortable discussing present actions. This study supports the usefulness of applying linguistic analysis to Reddit posts in an effort to understand opioid users. However, this study did not examine differences between pro- and antiopioid Reddit posts, which may further reveal attitudes among opioid users.

##### This Study

Little is known about how Reddit users post about opioids in web-based communities, the degree to which engagement occurs, and the themes present in pro- versus antiopioid posts. The aim of this study is to conduct a content analysis evaluating engagement and linguistic elements of pro- and antiopioid use posts on Reddit.

#### Methods

##### Study Design

We conducted a content analysis using the natural language processing of publicly available Reddit posts. This study was exempt from human subjects review by the University of Wisconsin-Madison Institutional Review Board.

##### Reddit Post Selection

We identified a sample of Reddit posts by searching for *opioid* on Reddit and clicked on the first subreddit, which also had the largest membership. This selection approach allowed us to replicate how an adolescent might naturally look for information about opioid use on Reddit.

Posts within this subreddit were sorted by the most recent, and the first 100 posts were pulled for analysis. Posts were included for analysis if they discussed opioid use and took a positive or negative stance regarding opioid use. Neutral posts were excluded from the analysis. To determine whether posts were pro- or antiopioid use, we developed a codebook based on previous research [45]. Pro-opioid posts mentioned usage of drugs, questions about usage and sourcing, and addiction without a stated intent or desire to recover. Antiopioid posts mentioned seeking help for recovery, withdrawal, and sobriety. One investigator (SG) categorized posts as positive or negative using a deductive approach. A second investigator coded a 19.61% (20/102) subsample of both Reddit (n=10) and Twitter (n=10) posts to calculate the interrater agreement. Interrater agreement was calculated as the percentage of Reddit and Twitter posts referencing each category, ranging between 85% (17/20) and 100% (20/20), with a mean of 92.7% (SD .05%)

##### The LIWC Program

Pro- and antiopioid posts were evaluated using the LIWC software described in the Introduction section of this compilation [28]. LIWC software scans text and calculates the percentage of text words that fall within a given dictionary. LIWC has been used in previous studies to evaluate publicly available text and understand differences in content and tone [29].

##### Measures

###### LIWC Dictionaries

The selected LIWC variables were aligned with the specific aims of our study. Previous research has shown that the results of opiate use include changes in physical and mental health, isolation from family and friends, and financial difficulties [46]. Therefore, we included variables related to tone, positive emotion, negative emotion, health, relationships, and focus (past, present, and future). The three focus variables show whether the text describes past events (*used*, *ago*), present events (*now*, *today*), or future events (*will*, *soon*). These variables were selected based on their relevance to opioid use [47]. Table 3 shows the full list of LIWC variables, with example words coded for those variables.

**Table 3:** Study 2—LIWC^a^ variables included with example words.

LIWC variable	LIWC example words
Tone	—^b^
Ipron	*It*, *to*, and *no*
Posemo	*Love* and *nice*
Negemo	*Hurt* and *ugly*
Anxious	*Worried*
Anger	*Hate* and *kill*
Sad	*Crying* and *grief*
Family	*Dad* and *aunt*
Friend	*Buddy* and *neighbor*
Body	*Cheeks* and *hand*
Health	*Clinic*, *flu*, and *pill*
Focus past	*Ago*, *did*, and *talked*
Focus present	*Today*, *is*, and *now*
Focus future	*May*, *will*, and *soon*
Work	*Job*, *major*, and *xerox*
Money	*Audit*, *cash*, and *owe*

^a^LIWC: Linguistic Inquiry and Word Count.

^b^Not available. The LIWC 2015 development manual does not provide example words for Tone.

###### Engagement

We collected data on how individuals may interact with pro- or antiopioid posts by logging the number of upvotes and comments on each post. The upvote feature on the Reddit platform is typically used as an indication of the support or approval of a post.

###### Data Collection Procedure

Data were collected on Reddit posts from July 11 to 12, 2020. Interrater agreement for positive posts was 80% (8/10). All posts were copied and pasted verbatim into Google sheets.

##### Analysis

Descriptive statistics were used to understand the engagement with pro- and antiopioid posts. A two-tailed *t* test was used to compare LIWC scores for each variable between pro- and antiopioid Reddit posts. Statistical significance was set at *P*<.05.

#### Results

##### Sample Characteristics

At the time of the analysis, this subreddit had approximately 116,000 members. A total of 100 posts were included in the analysis. All posts were dated between July 8, 2020, and July 26, 2020. In this sample, 65% (65/100) of the posts were pro-opioids, and 35% (35/100) were antiopioid. There were more comments on antiopioid posts (mean 14.88, SD 16.37) than on pro-opioid posts (mean 9.06, SD 12.22; *t_81_*=2.79; *P*=.05). Similarly, there were more upvotes for antiopioid posts (mean 41.67, SD 103.43) than for pro-opioid posts (mean 1.58, SD 1.98; *t_81_*=16.84; *P*=.002).

An example pro-opioid post was the following, “Hey guys, was just wondering based on your experiences, what is the best oxycodone brand.”

An example antiopioid post was the following:

Hi I’m new on here and I’m happy I have found a place to talk about my addiction, I really need help. I’ve been on 200 mg of Diazepam and 900mg of Codeine Phosphate. I am very scared I might overdose and I just want to be sober!

##### LIWC Results

The scores on the LIWC variable *Focus Present* were significantly higher for antiopioid posts (mean 15.58, SD 9.81) than for pro-opioid posts (mean 11.19, SD 5.79; *t_98_*=2.82; *P*=.02). The *Focus Future* variable was also significantly higher for antiopioid posts (mean 1.63, SD 1.86) than for pro-opioid posts (mean 0.83, SD 0.96; *t_98_*=2.84; *P*=.02). There were no other statistically significant differences between pro- and antiopioid posts (Table 4).

**Table 4:** Study 2—engagement and linguistic differences between pro- and antiopioid posts on Reddit.

Variable	Antiopioid (score), mean (SD)	Pro-opioid (score), mean (SD)	*P* value
Focus present	15.58 (9.82)	11.19 (5.80)	.02
Focus future	1.63 (1.86)	0.83 (0.97)	.02
Upvotes	41.67 (103.43)	1.58 (1.98)	.002
Comments	14.48 (16.37)	9.06 (12.22)	.05

#### Discussion

##### Principal Findings

The purpose of this study was to examine the differences between pro- and antiopioid posts on Reddit. We found that there were more comments and upvotes on antiopioid posts than on pro-opioid posts. Antiopioid posts were more likely to contain linguistic elements related to the present and future than pro-opioid posts. Our results show that there may be differences in how antiopioid and pro-opioid Reddit users relate to the present and the future. Our results further show that although pro-opioid posts may be more common in some Reddit communities, antiopioid users who post in these communities are likely to experience support (in the form of upvotes) and engagement (in the form of comments).

Most posts on the Reddit opioid community were pro-opioid. However, there were more upvotes for antiopioid content than for pro-opioid content. As the upvote feature is typically used to indicate support or approval, this finding suggests that users who wish to discuss the negative effects of opioid use may find support (in the form of upvotes) on Reddit. More frequently, upvoted posts are sorted to the top by the Reddit algorithm. Therefore, antiopioid posts, which received more upvotes in this study, may also be seen by more users of this subreddit.

There were also fewer comments under pro-opioid posts than antiopioid posts. Comments are a primary way for users to interact with each other in the community and may indicate engagement, support, and discussion. Our results suggest that people who share pro-opioid posts may be met with less conversation than those who share antiopioid posts. However, in this study, only the number and not the content of comments were evaluated. Future studies should examine whether comments on antiopioid posts are positive or negative.

Of the 16 LIWC variables measured, only the *Focus Present* and *Focus Future* variables were significantly different between pro- and antiopioid use posts. This finding is somewhat consistent with a previous study that found differences in time focus between anonymous and nonanonymous Reddit users [44]. We found that nonanonymous users used more words related to the past, which may be due to concerns about nonanonymously disclosing current or planned activities. In this study, a difference in focus was also identified. Words such as *today*, *now*, and *will* were more frequent in antiopioid posts than pro-opioid posts. It may be that antiopioid Reddit users are more likely to comment on the negative present or anticipated future outcomes associated with opioid use.

No other variables were significantly different between pro-opioid and antiopioid posts. This finding was surprising, as the variables were chosen based on outcomes of opioid use, and social media displays of drug use have been shown to reflect real-life use [41]. We had expected that antiopioid posts, for example, would describe negative emotions (*scared*) or describe the topics of family, friends, or financial problems [46]. It may be that pro-opioid users chose to focus on other aspects of drug use. For example, past research on e-cigarette use shows that Reddit conversations are dominated by conversations about how to access these products [48]. In contrast, antiopioid Reddit users may have chosen not to share negative outcomes related to health, family, or work.

##### Limitations

This study’s external validity was limited by the small sample size and the relatively short length of the period for which the posts were collected. Reddit does not provide demographics for its users, so we cannot confirm that the study participants were adolescents or young adults; however, research suggests that Reddit is more popular among young adults than any other demographic [43]. Therefore, young people are likely to encounter the pro- and antiopioid content described in this study.

##### Conclusions

This study was one of the first to examine the engagement and linguistic elements of opioid use in a popular subreddit. This study showed that there is still much that needs to be researched about how individuals engage in web-based communities about opiates. The findings of this study indicate that, except for verb tense, word usage in posts about opiates may not distinguish pro- from antiopioid Reddit users. Future studies should investigate how members of opioid discussion groups on Reddit and other social media interact with opioid-related content, including through likes or upvotes and in the comment sections. If Reddit’s algorithm can identify youths who misuse opioids, Reddit can suggest resources and assist its users in finding help.

## Study 3

*This study was prepared by Anjali Mathur and mentor Bradley Kerr*

### References to Positive and Negative Self-esteem on the Comments of Beauty-Related YouTube Videos: Content Analysis

#### Introduction

##### Self-esteem in Adolescence

Adolescence is the stage between 11 and 21 years of age, and it is a critical period for the development of self-esteem [49]. Self-esteem is defined by how one positively or negatively views oneself, and low self-esteem is associated with depression, anxiety, and suicidal ideation [50-53]. In adolescence, self-esteem is especially vulnerable to protective or harmful influences [54]. Therefore, it is important to understand influences on adolescent self-esteem, and social media may present one such influence. Previous studies have found that social media use is associated with lower self-esteem [55,56].

##### Social Media Influencers and Self-esteem

One way in which social media influences adolescents’ self-esteem is through social media influencers. Social media influencers are content creators with a large social media following. Individuals with low self-esteem are more likely to make upward comparisons between themselves and influencers [54,57]. Beauty-related YouTube content creators are influencers that share aspects of their personality, esthetics, and preferences, allowing viewers to relate to them [57]. Specifically, beauty-related YouTube content creators post videos that review and promote makeup products while also entertaining their viewers through sharing tutorials and trends [57,58]. Such content may potentially impact adolescent viewers’ self-esteem, given the focus on physical appearance and that YouTube is used by 77% of adolescent internet users [59]. These youths may seek advice on purchases, enjoy video entertainment, or watch videos to relax [60]. In addition, the popularity of beauty-related videos on YouTube has increased drastically as the annual viewership increased from 59 billion in 2016 to 169 billion in 2018 [58]. This large viewership suggests that these videos have the potential to reach and influence a wide audience, which may include many adolescents. Thus, it is important to understand the influence of viewing beauty-related YouTube content creators’ videos on adolescents’ self-esteem.

##### This Study

Previous studies have examined content shared by beauty-related YouTube content creators and identified methods creators use to gain followers and influence their viewers [57]. Beauty-related YouTube videos may affect the self-esteem of viewers, and it is possible that these viewers describe the effects on their self-esteem in comments on these videos. However, previous studies have shown that Instagram users may post positively toned comments while self-reporting negative effects on their body image [61]. Thus, the presence of self-esteem-related discussions in beauty-related YouTube content creators’ video comment sections and their tone remain unclear. The aim of this study was to examine the expression of and overlap between self-esteem and tone in comments on beauty-related YouTube videos.

#### Methods

##### Study Design

We conducted a content analysis of publicly available YouTube comments, which is described in the Introduction section of this compilation. This method allowed for the objective evaluation of conversations in beauty-related YouTube video comment sections. This study was exempt from human subjects review by the University of Wisconsin-Madison Institutional Review Board.

##### Search Strategy

We identified a sample of 6 beauty-related YouTube content creators whose content focuses on makeup. To identify beauty-related YouTube content creators likely to be viewed by adolescents, we used the search term *most popular makeup youtubers* in the Google search engine. The first four relevant results were reviewed, and beauty-related YouTube content creators who were represented on more than one website were selected [62]. Beauty-related YouTube content creators were included if, within their top 16 most recently posted videos, at least two videos included *makeup* or *palette* in the title of the video. For each beauty-related YouTube content creators, the two most recent videos including the word *makeup* or *palette* in the title were included in this study.

##### Comment Inclusion Criteria

The 20 most recent comments for each beauty-related YouTube content creator’s selected video were evaluated if they contained more than a username, were written in English, and included words (not just emojis). For comments that received responses, only the initial comments were included, not the responses.

##### Measures

###### Positive and Negative Self-esteem

We developed a codebook adapted from the Rosenberg Self-Esteem questionnaire to evaluate the presence of positive and negative self-esteem references [63]. The 10 items from the questionnaire were used to define positive and negative self-esteem references. The questionnaire included both positively and negatively framed statements. Each item was used for both positive self-esteem and negative self-esteem codes by reframing the statements. The positive self-esteem code was created using positively framed statements and by converting negative questionnaire items to positive statements. Similarly, the negative self-esteem category was developed using negatively framed statements and by converting the positive questionnaire items to negative statements. Table 5 shows the full definitions of positive and negative self-esteem.

**Table 5:** Study 3—codebook values and definitions.

Codebook values			Definition
**Self-esteem**			
	Positive	Satisfied with self, thinking they are good, having good qualities, feeling they are just like or better than others, feel proud of themself, feeling important, feeling worthy, respects themself, feels successful, and positive about self or life	
	Negative	Unsatisfied with self, thinking they are no good at all, feeling they have little/no good qualities, feeling nothing compared with others, having little to be proud of, feeling useless, feeling like a person of little worth, disrespectful to self, thinks they are a failure, and negative attitude toward self	
**Tone**			
	Positive	Good, pleasant, happy, joyful, contented, commenter offers a suggestion, impressed, love, gratitude, inspiration, fabulous, and constructive suggestions offered by commenters	
	Negative	Bad, unpleasant, sad, afraid, angry, dislike, out of control, boring, disgusted, hate, terrible, canceled, and corrections offered by commenters	

###### Positive and Negative Tone

We developed a codebook adapted from the Positive and Negative Experience Scale to evaluate tone [64]. The initial codebook was pilot tested and refined using the study data. The initial codebook defined positive tone using the following adjectives from the scale: good, pleasant, happy, joyful, and contented. Additional phrases suggesting a positive tone were added to the codebook after pilot testing, including adjectives (impressed, love, gratitude, inspiration, and fabulous) and constructive suggestions offered by commenters. Negative tone was also defined using adjectives from the Scale of Positive and Negative Experience, including bad, unpleasant, sad, afraid, angry, and dislike. Additional phrases suggesting a negative tone were added to the codebook after pilot testing, including adjectives (out of control, boring, disgusted, hate, terrible, and canceled) and corrections offered by commenters.

###### Audience Engagement

We recorded the number of likes, dislikes, comments, and views on each video, as well as the follower count for each beauty-related YouTube content creator’s channel.

##### Data Collection Procedures

Data were collected from each video, and comments were coded in October 2020. A second investigator coded a 10% (24/240) subsample of the comments. Interrater agreement was calculated for each codebook measure as the percentage of YouTube comments coded the same between the two investigators. Interrater agreement ranged between 88% (21/24) and 100% (24/24), with a mean of 95.8% (SD 0.06).

##### Analysis

Descriptive statistics were calculated to assess the prevalence of references to self-esteem and tone.

#### Results

##### YouTube Videos and Creators Characteristics

A total of 12 videos were included, two videos from each of the 6 beauty-related YouTube content creators. Likes per video ranged from 7000 to 634,000 with an average of 129,925.0 likes (SD 191,029.8), whereas dislikes ranged from 109 to 85,000 with an average of 14,538.4 dislikes (SD 29,260.2). Comments ranged from 424 to 37,724, with an average of 13,279.3 comments per video (SD 16,159.9). Views per video ranged from 107,437 to 10,168,197, with an average of 2,362,004.0 views (SD 3,211,374.3). The beauty-related YouTube content creators had a range of 4.8 million to 23.1 million subscribers to their channel.

##### Positive and Negative Self-esteem

A total of 240 comments were evaluated. Among these comments, 5.4% (13/240) reported positive self-esteem. An example of a positive self-esteem comment was:

*I’m not really a brand...yet.But, I do want to take over the world of vintage clothing. You’re an inspiration*. [beauty-related YouTube content creator’s name]

Of all the comments, 6.3% (15/240) referenced negative self-esteem. For example, a person commented:

I would be terrible friends with them, I don’t like make-up on me, I’m broke, I’m not popular, and I don’t keep up with trends.

No comments referenced both positive and negative self-esteem. Of the comments, 88.3% (212/240) did not refer to self-esteem.

##### Positive and Negative Tone

Among the comments evaluated, 65.4% (157/240) exhibited a positive tone. An example of a comment with a positive tone included, “HE LOOKS GOOD WTF.” Of all comments, 17.5% (42/240) displayed a negative tone. For example, a person said, “You look like You did an absolutely terrible kawaii Makeup.” Of the comments evaluated, 3.3% (4/240) exhibited both positive and negative tone. For example, a commenter wrote:

As a victim of the One Chip Challenge...DON’T DO IT! hahahahaha!!! I am a wuss and totally had a panic attack...I did get some pretty funny footage though LMAO.

Of all comments, 18.7% (45/240) displayed neither positive nor negative tone.

##### Overlap in Self-esteem and Tone

Among the comments that referenced positive self-esteem, 100% (13/13) reflected a positive tone. Positive self-esteem was referenced in 8% (1/13) of positive-toned comments. Among the comments that referenced negative self-esteem, 54% (7/13) reflected a positive tone, and 69% (9/13) reflected a negative tone. Negative self-esteem was referenced in 4.5% (7/157) of positive-toned comments and 21% (9/42) of negative-toned comments.

#### Discussion

##### Principal Findings

Through this study, we found that few comments on beauty-related YouTube videos referenced self-esteem. Among the self-esteem comments, a similar number of positive self-esteem and negative self-esteem comments were observed. Most comments on the sampled beauty-related videos showed a positive tone.

The first main finding was that 11.7% (28/240) of comments referenced self-esteem. A possible explanation for this finding is that many feel uncomfortable discussing self-esteem on the web. Previous research suggests that social media users feel pressured to only post what would make them, as an individual, look good [65], which may influence viewers to avoid commenting negatively about their self-esteem. It is also possible that the viewer avoids commenting on their self-esteem if the rest of the comment section appears to include few comments referencing others’ self-esteem. Finally, it may be that few viewers experienced changes in their self-esteem.

Our second main finding was that there were a similar number of comments that referenced positive and negative self-esteem. One possible reason for this finding is that beauty-related YouTube videos can impact a viewer’s self-esteem both positively and negatively. This finding aligns with a previous study that suggested that YouTube videos can both hurt and help the viewer’s self-esteem based on their understanding and relatability to the beauty-related YouTube content creators [66]. However, another possibility is that YouTube users comment on their existing self-esteem levels when viewing YouTube content and may not be influenced by the videos themselves. Therefore, it is not clear what prompts positive and negative self-esteem discussions within the comments of beauty-related YouTube content creator videos.

An additional finding was that most comments displayed a positive tone. This aligns with previous studies that suggest that beauty-related YouTube content creators build their platform to spread positivity [67] and that viewers are more likely to comment with supportive material [68]. A positive tone may indicate a positive viewing experience and positive influences on self-esteem; however, research conducted on Instagram suggests that users may comment positively on a post but self-report negative effects on their own body image [61]. Thus, it is possible that a positive tone may not reflect a positive change in the viewer’s self-esteem. In addition, it is possible that the positive tone conveyed in the comment section may influence the viewer to avoid commenting negatively or commenting on their self-esteem.

##### Limitations

One limitation of this study was that self-reported self-esteem was not measured. Future work should examine the self-reported self-esteem of adolescents who view beauty-related videos on YouTube. In addition, those who comment on YouTube videos may not be representative of all viewers of the video, and it is not clear how findings generalize across all video viewers. In an attempt to review a broader set of viewer comments, multiple beauty-related YouTube content creators were included in this study. Similarly, the ages of the commenters are unknown; thus, it is possible that there are comments not shared by adolescent viewers. Nonadolescent viewers may be less vulnerable to negative self-esteem as a result of watching beauty-related YouTube content creators. However, as there are a large number of adolescent viewers on YouTube [69], it is possible that many comments were posted by adolescents. Furthermore, we excluded emojis from our coding processes as emojis may have multiple interpretations based on their context and may be difficult to code objectively.

##### Conclusions

Despite these limitations, our study has several important implications. Given the high frequency of comments on beauty-related YouTube videos with a positive tone, coupled with the low frequency of self-esteem disclosures, it is possible that adolescents would feel uncomfortable discussing negative effects on their self-esteem in this web-based environment. Future studies should examine avenues in which adolescents discuss their self-esteem in connection with beauty-related videos on YouTube. Future research should also explore effective approaches for parents to engage in conversations with their children about beauty-related videos on YouTube. Furthermore, some self-esteem references in comments suggests the possibility that these videos could affect adolescents’ self-esteem. Further studies should investigate the effects of beauty-related YouTube videos on viewers’ self-reported self-esteem.

## Study 4

*This study was prepared by Ethan Kaji and mentor Maggie Bushman*

### Comparing the Representation of Depression on Reddit and Twitter Social Media Platforms: Content Analysis Study

#### Introduction

##### Social Media and Depression

The most prominent mental illness affecting adolescents is depression, with 4%-5% of adolescents impacted worldwide each year [70]. If depression symptoms are not treated, it can lead to recurrence later in life [70]. The most extreme cases of depression can also lead to suicide, a major cause of death among adolescents [71,72]. Oftentimes, people with depression will post their feelings and inner thoughts on social media platforms, giving others a chance to respond and support them [73]. A study of Facebook accounts found that participants who showed symptoms of depression on the web also self-reported symptoms of depression [74]. Previous research has evaluated how depression is discussed on social media and has investigated ways to identify users with depression [75]. Computer algorithms can detect depression-related content in posts on social media with an accuracy of more than 90% [75]. Being able to consistently identify symptoms of depression on social media could lead to earlier treatment for adolescents.

##### Reddit Versus Twitter

Social media platforms, such as Reddit and Twitter, provide spaces for adolescents to discuss the triumphs and tribulations of their daily lives on the web, including personal information about their school, family, and friends [76]. Reddit is a social media platform divided into distinct communities to foster discussions among users [42]. These communities, often called subreddits, are created and moderated by users rather than the Reddit platform itself. Users can post specific subreddits, and others can respond by continuing the thread. Previous research has suggested that an anonymous platform such as Reddit encourages the discussion of more emotional or sensitive information [77].

In contrast to Reddit, Twitter is a platform designed around short statements made by users to convey information in real time [78]. A study from 2017 found that users who tweet about mental health do so because Twitter provides a sense of community, a safe space for expression, and means of coping [79]. Users also use Twitter to spread awareness. Little is known about the differences in how adolescents talk about their depression on an anonymous, forum-based platform such as Reddit compared with a personal, newsfeed platform such as Twitter. Therefore, this study aims to compare depression posts on Reddit, a forum-based platform, and Twitter, a newsfeed platform, to understand how users talk about depression on the web.

#### Methods

##### Study Design

We conducted a content analysis of publicly available Reddit and Twitter posts on October 28, 2020, to determine the number of posts that showed symptoms of depression and other themes related to youth. Reddit posts were defined as the first posts in the depression subreddit *r/depression*. Twitter posts were defined as posts that used the hashtag #depression. This study was exempt from human subjects review by the University of Wisconsin-Madison Institutional Review Board.

##### Search Strategy

We identified a national sample of publicly available Reddit and Twitter posts. Reddit posts were taken directly from the subreddit r/depression. Posts were evaluated under the *new* tab to view a wide range of recent posts, rather than only the most popular. Twitter posts were collected using the search term #depression. The *latest* tab was used on Twitter to ensure a variation of posts. Using the search terms r/depression and #depression, we sought to replicate search strategies adolescents would use when discussing depression on social media.

##### Post Inclusion Criteria

Reddit posts were included if they were the most recent posts in r/depression. Twitter posts were included if they were some of the most recent posts made with #depression. On Twitter, posts were included if they contained content in addition to hashtags. Posts with pictures were considered on both platforms. However, posts written in a language other than English, with videos, and made by accounts that stated that they were bots were not considered. Duplicate posts or identical posts published by either the same or different accounts were excluded from the sample.

##### Measures

An investigator categorized Reddit and Twitter posts into major topic categories using deductive and inductive approaches. The investigator reviewed each post to determine if symptoms of depression and youth topics were discussed. Open coding was used to generate codes for promotional posts and medical topics, as these themes emerged while coding these posts. A codebook that used the Diagnostic and Statistical Manual of Mental Disorders (DSM)-IV criteria from a previous study was adapted to determine whether posts contained symptoms of depression [74]. A full list of codes and their prevalence is shown in Table 6.

Each post included in the study was coded for the following categories: at least one symptom of depression; at least one youth topic; promotional posts; and medical topics. A symptom of depression was defined by mentioning at least one of the nine DSM-IV criteria, including categories such as *depressed mood*, *insomnia or hypersomnia*, and *recurrent thoughts of death*. A complete list of DSM-IV categories can be found in Table 6. Youth topic variables included specific references to school, family, and social activities. Youth topic variables were included to discern whether adolescents were likely posting using #depression and r/depression. Adolescents might also discuss topics such as school, family, and social activities. Promotional posts included posts endorsing any material or content. Examples included promotions for blogs on exercise, books on veterans, and seminars on meditation. Finally, medical topics included medical references, such as medications, hospital visits, and therapy. An investigator coded the samples of the Reddit and Twitter posts. A second investigator coded a 10% (n=40) subsample of both Reddit (n=20) and Twitter (n=20) posts to calculate the interrater agreement.

**Table 6:** Study 4—codebook categories, definitions, and percent of posts.

Categories	Definitions and examples	Reddit posts referencing code (n=53), n (%)	Twitter posts referencing code (n=49), n (%)
Depressed mood	Sad, empty, crying, tearful, distressed, and down (unless context clarifies otherwise)	47 (89)	8 (16)
Decreased interest or pleasure in activities or anhedonia	Not having fun, do not feel like doing anything, giving up, lack of purpose, and not caring	17 (32)	0 (0)
Changes in weight or appetite	No appetite, do not feel like eating, cannot stop eating, and eating everything in sight	0 (0)	0 (0)
Insomnia or hypersomnia	Tired, exhausted, sleepy, need a nap, easily tired, not sleeping well, restless sleep, cannot sleep, cannot get to sleep, falling asleep in an unusual place, and insomnia	4 (8)	0 (0)
Agitation or slowing down of movement	Inability to sit still or feeling slow	2 (4)	1 (2)
Fatigue or loss of energy	Cannot get anything done and cannot get motivated due to fatigue	8 (15)	1 (2)
Feelings of worthlessness or guilt	Feel guilty or worthless, “I am stupid,” “I’m not cool,” “I’m average,” “I’m crazy” or “I’m insane,” regretting something, being a failure, or failing	20 (38)	1 (2)
Difficulty concentrating or indecisiveness	Cannot study, cannot finish work, cannot concentrate because of emotion, cannot decide on something, do not feel like deciding, cannot make up your mind, and not knowing what to do	7 (13)	0 (0)
Recurrent thoughts of death	Thinking of ways to commit suicide, references to jumping, referencing death of self, and thinking about death	23 (43)	1 (2)
School	Specifically references school-related events, goals, and extracurricular activities	13 (25)	0 (0)
Family	Specifically references parents, guardians, siblings, and other family members	20 (38)	4 (8)
Social activity	Specific references to social life	20 (38)	2 (4)
Promotional content	Promoting any material or content, that is, blogs, studies, and podcasts	0 (0)	26 (53)
Medical topics	Medical content appears within the post	15 (28)	9 (18)

##### Data Collection Procedures

Data were collected on each Reddit and Twitter feed on October 28, 2020. Interrater agreement was calculated as the percentage of Reddit and Twitter posts referencing each category, ranging between 85% (34/40) and 100% (40/40), with a mean of 92.7% (SD .05%)

##### Analysis

Descriptive statistics were calculated for all measures. A chi-square test was used to analyze the relationship between each platform and the following main categories: the proportion of mentions of at least one symptom of depression; at least one youth topic; promotional content; and medical topics. Statistical significance was set at *P*<.05. The average DSM-IV scores were calculated by calculating the total number of DSM-IV symptoms that appeared on each platform and dividing by the number of posts collected from each of them.

#### Results

##### Overview

A total of 53 posts were selected from the subreddit r/depression. At the time of coding (October 2020), this subreddit had approximately 700,000 members. A total of 49 tweets were selected from the social media platform Twitter.

##### Symptoms of Depression and Youth Codes

We found that 92% (49/53) of posts on Reddit and 24% (12/49) of posts on Twitter contained at least one symptom of depression (*P*<.001). *Depressed mood* was referenced by 89% (47/53) of Reddit posts and 16% (8/49) of Twitter posts. Common phrases that denoted *depressed mood* included terms such as *crying* and *sad*. *Recurrent thoughts of death* was the second most common depression symptom and was referenced by 43% (23/53) of Reddit posts and 2% (1/49) of Twitter posts. The average Reddit post received a DSM-IV score of 2.4, whereas the average Twitter post received a DSM-IV score of 0.2. Similarly, we found that the percentage of posts that mentioned at least one youth code was 62% (33/53) on Reddit and 10% (5/49) on Twitter (*P*<.001).

##### Promotional and Medical Codes

We found that promotional content appeared in 0% (0/53) of Reddit posts and 53% (26/49) of Twitter posts (*P*<.001). We also found that the percentage of posts referencing medical topics was 28% (15/53) on Reddit and 18% (9/49) on Twitter (*P*=.24). For the full results describing the codes and frequency, see Table 6.

#### Discussion

##### Principal Findings

This study compared Reddit and Twitter posts discussing depression. Results suggested that the discussion of depression was significantly more common on Reddit than on Twitter, with 92% (49/53) of Reddit posts and 24% (12/49) of tweets mentioning at least one symptom of depression. Furthermore, Reddit posts received an average DSM-IV score of 2.4, whereas Twitter posts received an average DSM-IV score of 0.2. This difference in expression fits with existing literature that adolescents are more or less willing to reveal certain emotions based on the type of social media platform they are using [80]. Our results also suggested that Reddit posts may be more likely to be posted by adolescents, with 62% (33/53) of posts on Reddit referencing and discussing at least one youth code compared with 10% (5/49) of posts on Twitter mentioning such subjects. Another important finding is that Twitter posts were significantly more likely to contain promotional content than Reddit posts. None of the Reddit posts investigated contained promotional content. In comparison, 53% (26/49) of tweets contained promotional content.

Our findings may help understand the potential differences in discussions of depression on Reddit and Twitter. These findings suggest that users are more likely to elaborate on their experiences with depression when posting on the subreddit r/depression rather than with #depression on Twitter. This may be because of the anonymity of r/depression [77].

Reddit communities are also moderated by users who volunteer as *moderators*. Moderating powers include the ability to remove posts, comments, and users from the community. It is possible that the heavy moderation of r/depression by users compared with Twitter encourages others to be more open with their posts on Reddit [42]. A moderated Reddit thread could become a safe place for adolescents to discuss mental health and depression. They might find it comforting that a moderator would be able to remove hurtful or harmful posts or users from the subreddit. Both Reddit and Twitter have features to report posts or messages; however, having a moderator could take the burden of reporting off the shoulders of adolescents and onto a third party. Another possibility could be that subreddit moderators can remove posts that do not align with the subreddit’s mission, such as promotional content, resulting in longer posts in which users can expand on their experiences with depression.

The finding that more Reddit posts referenced at least one youth code compared with Twitter could suggest that there is a wider audience of adolescents using r/depression to discuss mental health than Twitter. This finding is consistent with the demographics of each platform. In recent years, Reddit’s age demographic has been trending younger, with 21% of users aged between 18 and 24 years [81]. Conversely, Twitter’s age demographic has been trending older, with 28% aged between 35 and 49 years [82].

Finally, the larger percentage of posts with promotional content on Twitter than on Reddit suggests that tweets with symptoms of depression are diluted by tweets from other topics such as promotions for blogs on exercise or seminars on meditation, sometimes unrelated to clinical depression. The promotional nature of the content on Twitter could be why references to youth-based topics such as school, family, and social activity were more common on Reddit than on Twitter. There is a chance that the promotional use of #depression could deter adolescents experiencing symptoms of depression from discussing their experiences on Twitter.

##### Limitations

The sample of Reddit and Twitter posts included in this study was small. However, patterns in the data were still identified. Another limitation was the timeframe for the collection of posts. All posts were collected during the COVID-19 pandemic, where stay-at-home orders and isolation could have influenced the data. Although this limitation might skew the number of posts that show at least one symptom of depression, it should affect both platforms equally without affecting the overall comparison.

##### Conclusions

Future studies should consider investigating other moderated communities for users experiencing depression, such as Facebook groups. Future studies should also consider comparing Instagram, a photo-based social media platform, with Reddit, a forum-style platform, which could yield important information on how the inclusion of photographs makes users more or less likely to discuss mental health topics. Comparisons should also be made with platforms that target foreign audiences, as both Reddit and Twitter have a majority of US users. Further understanding of how adolescents discuss depression on the web could help inform guidelines for social media support communities. Although computer algorithms could be used to detect posts about depression, supporting web-based communities where human detection is taking place could provide adolescents with the resources they need to get help. It could also help clinicians understand youth experiences with depression, what web-based resources they access for support, and lead to earlier treatments.

## Study 5

*This study was prepared by Owen Tsao and mentor Anna Jolliff*

### Mental Health Sentiments in the Comment Sections of Climate Change Posts on Instagram: Content Analysis

#### Introduction

##### Climate Change and Adolescent Mental Health

Climate change can result in mental illnesses, such as depression, anxiety, and posttraumatic stress disorder [83]. Climate change can impact mental health directly through exposure to traumas, such as forest fires and floods, and indirectly, as people hear news about climate change and its associated deaths [83,84]. The effects of climate change will disproportionately affect the youth and young people. Some of the decisions and mistakes made by previous generations (some necessary, some not) have, in turn, led to the ill effects of climate change. It is the younger and future generations who will have to answer for the mistakes of the past and live a greater proportion of their lives in a steadily degrading environment [85]. However, young people also have the ability to advocate against climate change.

##### Climate Change Advocacy on Social Media

Young people can advocate against climate change through new technological developments. Currently, the younger generation uses social media as a context to promote climate change activism [86,87]. Research shows that climate change advocacy on social media promotes knowledge and behavioral changes around climate change [88]. Social media *personalizes* the issue of climate change by adding photographs, conveying information through friends, and catering to users’ preferences for receiving information [88]. However, it is unknown whether climate change advocacy on social media is associated with depression or anxiety, as viewers are exposed to negative news and messages. Furthermore, research on climate change advocacy on social media disproportionately focuses on Twitter, excluding popular sites such as Instagram [89]. It is crucial to study Instagram because it is one of the most popular social media platforms among adolescents [90].

##### This Study

Previous studies have confirmed the negative mental health effects of climate change and studied climate change activism on social media. However, it is unknown whether positively versus negatively framed posts receive more engagement in the form of likes, comments, or followers on the posters’ accounts. It is also unknown whether comment sections of positively and negatively framed posts include sentiments consistent with depression, anxiety, or positive affect. The aim of this study is to evaluate whether positively or negatively framed climate change Instagram posts receive more engagement and whether their comment sections demonstrate sentiments consistent with depression, anxiety, and/or positive affect.

#### Methods

##### Study Design

We conducted a content analysis of publicly available, positively and negatively framed climate change Instagram posts to understand whether sentiments consistent with depression and anxiety as well as sentiments consistent with positive affect could be found in their comment sections. This study was exempt from human subjects review by the University of Wisconsin-Madison Institutional Review Board. We selected Instagram because many adolescents actively use it. Although we could not confirm that commenters were adolescents, 37.1% of Instagram users are aged between 13 and 24 years, and Instagram is the most widely used social media platform among American teens [90,91].

##### Search Strategy and Post Eligibility

Our goal was to identify a sample of positive and negative posts on Instagram to evaluate the comments on these posts. We chose to search for posts using a hashtag page instead of a single Instagram profile so that we could see a variety of posts from different users. We performed a series of pilot tests to identify the most popular hashtags. From these pilot tests, we selected the hashtag #climatechange, as it is highly popular and contains a mix of positively and negatively framed posts on climate change. Next, we identified a sample of both positively and negatively framed posts by opening Instagram and searching using the hashtag #climatechange. Posts were sorted by the *most popular* feature on Instagram and considered for inclusion in chronological order, with the most recent viewed first to replicate how an adolescent might come across such posts.

**Figure 1:**
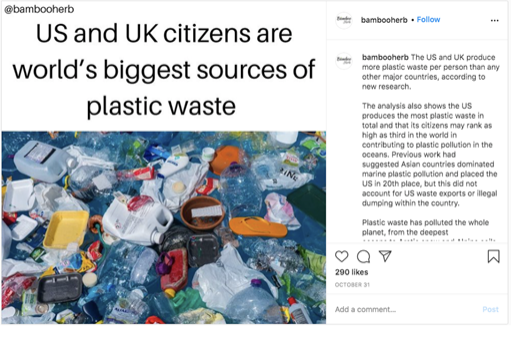
Example of a negatively framed climate change post.

**Figure 2:**
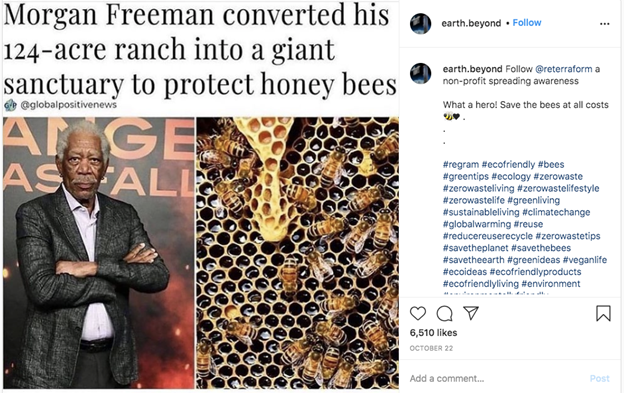
Example of a positively framed climate change post.

We defined a negatively framed post as a post that expressed a negative event or consequence of climate change (Figure 1). Inversely, a positively framed post was not one that advocated against climate change but rather gave good news about climate change, provided a solution, or was explicitly uplifting (Figure 2). Each selected post had to be identified as *positive* or *negative* by two investigators (OT and AJ) to be eligible for comment evaluation. Posts for which two investigators did not reach the same verdict, neutral posts, and duplicate posts were not included. Posts with fewer than five comments were excluded. The top five comments below each post, regardless of whether they earned a code, were included in the analyses.

##### Measures

###### Audience Engagement

We documented the number of likes and comments on each post. We also documented the number of followers for each account that shared a post.

###### Depression and Anxiety

We developed a codebook adapted from the DSM-V criteria to define criteria for sentiments consistent with depression and anxiety [92]. Emojis that expressed sentiments consistent with depression or anxiety were added inductively. For example, a crying emoji reflected the *sad* or *tearful* sentiment within depression, and the *nervous* emoji reflected the *worry* or *feeling on edge* sentiment of anxiety (see Table 7 for the full codebook).

**Table 7:** Study 5—codebook and example comments.

Sentiment type	Example sentiments	Emojis added during coding
Depression	Depressed mood, tearful, sad, empty, hopeless, irritable, less interest or pleasure in doing things, decrease or increase in appetite, too much or too little sleep, fatigue or loss of energy, and worthlessness or guilt	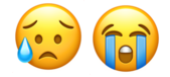
Anxiety	Anxiety; worry; restlessness; feeling on edge; easily fatigued; difficulty concentrating; mind going blank; irritability; difficulty falling or staying asleep; and distress or impairment in social, occupational, or other areas	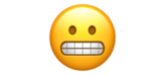
Positive affect	Determined, enthusiastic, excited, inspired, interested, proud, strong, happy, joyful, cheerful, lively, energetic, proud, confident, bold, and daring	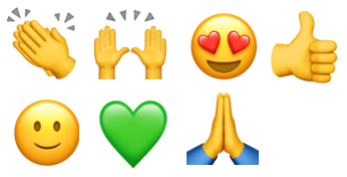

###### Positive Affect

We developed a measure of positive affect based on sentiments consistent with positive affect items from the Positive and Negative Affect Schedule–expanded form [93]. Emojis that expressed sentiments consistent with positive affect were added inductively. For example, the *applause* emoji was considered to reflect the *enthusiasm* sentiment within positive affect (see Table 7 for the full codebook).

##### Procedures

Data were collected for each post from October 29, 2020, to November 2, 2020. An investigator (OT) coded all comments using a combination of deductive and inductive approaches. A second investigator (AJ) coded a 10% (10/100) subsample of comments. Interrater agreement was calculated as the percentage of comments on which both coders agreed (9/10, 90% of all comments).

##### Analysis

Two-sided *t* tests were used to understand differences in the number of likes and comments between positively and negatively framed posts and the number of followers of accounts that shared positively versus negatively framed posts. Statistical significance was set at *P*<.05. Descriptive statistics were used to describe the prevalence of mental health sentiments in the comments.

#### Results

##### Post Characteristics

A total of 100 comments on climate change posts were selected from Instagram, with 50 comments coming from 10 positively framed posts and 50 comments coming from 10 negatively framed posts across 18 separate accounts. The average number of likes on positive posts was 5591.7 (SD 5435.2), whereas negatively framed posts had an average of 3400.3 likes (SD 4292.1), but this difference was not statistically significant (*t_18_*=1.00; *P*=.33). Positively framed posts’ average number of comments was 60.2 (SD 47.8), whereas the average number of comments on negatively framed posts was 74.8 (SD 72.3), and this difference was not statistically significant (*t_18_*=0.53; *P*=.60). Positive accounts had a range of 4125-342,000 followers, whereas negative accounts had 43,200-591,000 followers. Positively framed post accounts averaged 81,263.3 (SD 112,940.5) followers, whereas negatively framed post accounts had an average of 155,120 followers (SD 175,216.3). This difference was not statistically significant (*t_18_*=1.12; *P*=.27).

##### Mental Health Sentiments in Comments

Of the 100 comments, 17 (17%) referenced sentiments consistent with depression. An example comment was, “Yes we are and Shame on us.” Furthermore, 5% (5/100) referenced sentiments consistent with anxiety; an example was, “You’re *[sic]* weekly posts always make me feel so much better about my eco anxiety.” This post was also coded for positive affect. Finally, 32% (32/100) referenced sentiments consistent with positive affect, and an example was, “Good work 
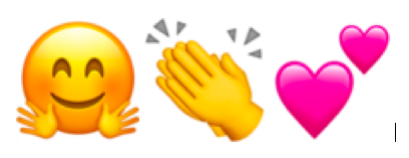
‼” Table 8 shows the frequency of depression, anxiety, and positive affect sentiments in comments under positively and negatively framed climate change posts.

**Table 8:** Study 5—frequency of mental health sentiments in comments of positively and negatively framed climate change posts (N=100).

Sentiment type	Positive post comments, n (%)	Negative post comments, n (%)
Depression	2 (2)	15 (15)
Anxiety	2 (2)	3 (3)
Positive affect	26 (26)	6 (6)

#### Discussion

##### Principal Findings

This study examined positively and negatively framed climate change posts and their comment sections on Instagram. Overall, both positively and negatively framed posts received thousands of likes and an average of >50 comments. There were no statistically significant differences between the number of likes or comments on positively versus negatively framed posts. This finding suggests that climate change posts can reach thousands of adolescents and positively influence young people, raising awareness without negative mental health consequences.

First, we found that all posts received thousands of likes and comments, and the accounts posting content all had thousands of followers. However, none of these numbers were significantly different between positively and negatively framed climate change posts. This finding suggests that climate change posts have a broad reach and receive engagement regardless of whether their message is positive or negative. Previous research shows that positive climate change messages may inspire action, whereas negative messages can result in passivity or helplessness [94,95]. Instagram should continue to allow both positively and negatively framed climate change posts on their platforms, as long as they are credible news statements, but should censor videos of murder, self-harm, violence, and other triggering content. As there was an equal engagement in positive and negative posts in this study, climate change advocates may wish to focus on sharing positively framed climate change posts.

Second, out of 100 comments, we found that 17 referenced sentiments consistent with depression, and 5 were related to anxiety. There was a total of 18 references to depression and anxiety under negatively framed posts and a total of 4 references under positively framed posts. This finding is consistent with previous research that noted a negative association between climate change and mental health [83,84]. However, only 22 sentiments were consistent with depression and anxiety in this study. We hypothesize that viewers may have been hesitant to share their true feelings on the internet, possibly because Instagram is not anonymous, and people may be nervous about expressing what seems like political views.

Finally, we found that 32% (32/100) of the comments referenced sentiments consistent with positive affect. This is a promising finding, as it suggests that climate change posts may inspire positive affect in some viewers. It is also possible that individuals are more likely to express positive opinions on social media, perhaps to seem more likable.

##### Limitations

We were limited by the small sample size of only 20 posts and 100 comments. In a larger sample, using a greater number of posts, it is likely that our numbers would be more generalizable. Furthermore, although we looked at the most recent comments on negatively and positively framed posts, responses to comments were not investigated. Another interesting challenge we encountered was the use of emojis in comments. It was surprising to see the number of comments that strictly used emojis, and interpretation may have been more subjective than the interpretation of words. Finally, we focused only on #climatechange. Searching for a larger variety of hashtags may have yielded different results.

##### Conclusions

This study has implications for the display of mental health sentiments in comment sections. Many comments showed sentiments of positive affect. Future research should aim to understand whether exposure to positively framed climate change posts positively affects mood and activism. Although this study focused on climate change advocacy on Instagram, future studies should examine climate change advocacy on other social media platforms. The high engagement in climate change advocacy Instagram pages in this study and the many positive messages may show the eagerness of the adolescent generation to approach climate change issues. Instagram should therefore be used as a platform to raise awareness on climate change issues as adolescents commonly use it, and this population is imperative for addressing climate change.

## Abbreviations

DSM: Diagnostic and Statistical Manual of Mental Disorders
LIWC: Linguistic Inquiry and Word Count
SRS: Summer Research Scholars
WHO: World Health Organization
